# Impact of chrysosplenetin on the pharmacokinetics and anti-malarial efficacy of artemisinin against *Plasmodium berghei* as well as in vitro CYP450 enzymatic activities in rat liver microsome

**DOI:** 10.1186/s12936-015-0929-3

**Published:** 2015-11-04

**Authors:** Shijie Wei, Hongyan Ji, Bei Yang, Liping Ma, Zhuchun Bei, Xiang Li, Hongwan Dang, Xiaoying Yang, Cheng Liu, Xiuli Wu, Jing Chen

**Affiliations:** School of Pharmacy, Ningxia Medical University, 1160# Shengli Street, Xingqing District, Yinchuan, 750004 People’s Republic of China; Institute of Clinical Pharmacology, General Hospital of Ningxia Medical University, Yinchuan, People’s Republic of China; Institute of Epidemic Disease, Academy of Military Medical Sciences, Beijing, People’s Republic of China

**Keywords:** Chrysosplenetin, Artemisinin, Pharmacokinetics, Anti-malarial efficacy, Rat liver microsome, CYP3A

## Abstract

**Background:**

Artemisinin (ART) is an efficacious and safe anti-malarial drugs but has low oral bioavailability and auto-induction profiles during multiple dosing. The pharmacokinetic disadvantages have been found to partially depend on the induction of cytochrome P-450 enzymes by ART and resulted in the therapeutic failure due to insufficient drug levels. The present study, therefore, investigated the impacts of chrysosplenetin (CHR), a polymethoxylated flavonoid from *Artemisia annua*, on the pharmacokinetics and the anti-malarial efficacy of ART against *Plasmodium berghei*. The inhibition of CHR on enzymatic activity of CYP1A2, CYP2A, CYP2C19, CYP2D6, CYP2E1, and CYP3A in rat liver microsome was also investigated. IC50, Km, Ki, and inhibitory type of CHR were respectively calculated.

**Methods:**

Twenty rats were randomly divided into four groups and received three-day oral doses of ART in absence or presence of CHR (in ratio of 1:0, 1:1, 1:2, and 1:4, respectively). Plasma samples were separately harvested for ART pharmacokinetics analysis using a valid liquid chromatography tandem mass spectrometric (LC–MS/MS) method. Female Kunming mice were inoculated by *P. berghei* K173 strain and pre-exposed to three-day oral administration of ART with or without CHR as pharmacokinetics protocol. Giemsa staining method was applied to calculate percent parasitaemia (%) and inhibition (%). In vitro rat liver microsomal model was employed to elucidate the inhibitory effect of CHR on CYP1A2, CYP2A, CYP2C19, CYP2D6, CYP2E1, and CYP3A.

**Results:**

The AUC_0–t_, C_max_, and *t*_1/2_ of ART increased significantly (*P* < 0.05 or *P* < 0.01) as well as declined CLz (*P* < 0.05 or *P* < 0.01) after three-day oral doses of ART in presence of CHR (1:2) when compared with ART alone. Also, parasitaemia (%) remarkably attenuated 1.59 folds with 1.63-fold augmented inhibition (%) when the ratio between ART and CHR reached 1:2. CHR itself had no anti-malarial efficacy (*P* > 0.05). CHR inhibited in vitro activity of CYP1A2 and CYP2C19 (*P* < 0.01, IC_50_ = 4.61 and 6.23 μM) in a concentration–response manner. The inhibition did not emerge on CYP2E1 and CYP3A until the CHR concentration exceeded 4.0 μM (*P* < 0.01, IC_50_ = 28.17 and 3.38 µM). CHR has no impact on CYP 2A and CYP2D6 (*P* > 0.05). The inhibition types of CHR on CYP1A2 and CYP3A belonged to noncompetitive and uncompetitive, respectively.

**Conclusions:**

Co-administration of ART with CHR in ratio of 1:2 achieved a synergic anti-malarial effect partly because of the noncompetitive or uncompetitive inhibition of CHR of drug-metabolism enzymes, especially CYP3A which is closely related to the auto-induction of ART.

## Background

**Fig. 1:**
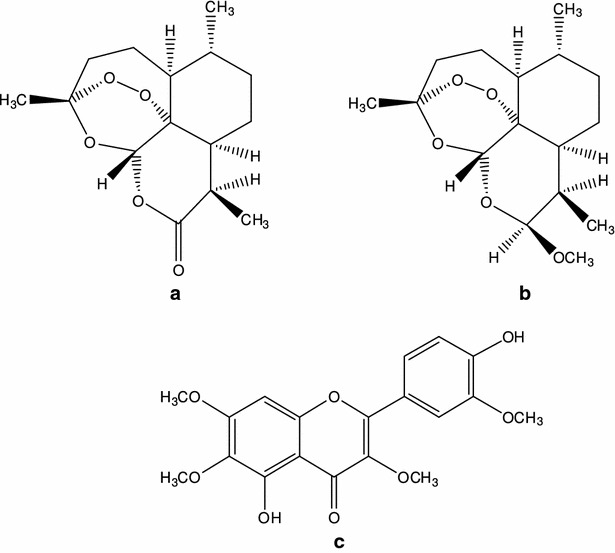
Structure of ART (**a**), ARM (**b**), and CHR (**c**)

Artemisinins (ART) (Fig. 1a) anti-malarial drugs belong to the sesquiterpene lactone family containing the specific endoperoxide bridge. They have been successfully used for more than two decades in the clinical treatment of malaria in regions with multi-drug resistant *Plasmodium falciparum* [1]. Up to now, ART and its semisynthetic derivatives are still the most important anti-malarial drugs available and ART-based combination therapy (ACT) has been recommended worldwide as first-line treatment for falciparum malaria since 2006 [2, 3]. Despite its widespread use, ART has very unusual pharmacokinetic properties with saturable first-pass hepatic metabolism and time-dependent pharmacokinetics during repeated oral administration [4–6]. ART, therefore, has very low oral bioavailability, merely 8–10 %. The auto-induction of both phase I and phase II metabolism of ART was demonstrated to be present in healthy Chinese subjects after a recommended two-day oral dose of ART-piperaquine probably due to the induction of CYP2B6 and CYP3A4 enzyme activity [7]. It was reported that ART in vitro metabolism was mediated primarily by CYP2B6, with a minor contribution from CYP2A6 and CYP3A4 [8]. El-Lakkany et al. [9] found that coadministration of grapefruit juice with artemether (150 mg/kg) eliminated eggs and granulomatous reactions and achieved complete protection of the host from damage induced by schistosomal infection because of the inhibitory effects of grapefruit juice on CYP450 and cyt b5.

Literature has shown that several polymethoxyflavonoid constituents from *Artemisia annua*, such as artemetin, casticin, chrysosplenetin (CHR, Fig. 1c) and chrysosplenol D may contribute to the activity of ART against *P. falciparum* [10, 11]. However, the mechanism of action has not yet been well defined. Generally, most flavonoids have an inhibitory effect on CYP450 enzymes and the aglycones have a stronger inhibition than glycosides [12, 13]. CHR has been previously enriched from the industrial wastes of ART about 1 g (over 98 % purity) and a China National Invention Patent (ZL201210093926.0, China) has been awarded. Structure of CHR was identified by ^1^H-NMR, ^13^C-NMR and 2D-NMR [14–19]. The present study was designed to investigate the impact of CHR on the pharmacokinetics and the anti-malarial efficacy of ART against *Plasmodium berghei*. Further, the inhibition of CHR on in vitro CYP450 enzymatic activity was also elucidated. It was demonstrated here that CHR increased the AUC, C_max_, and *t*_1/2_ of ART along with a decreased CLz and enhanced its in vivo anti-malarial efficacy against malarial parasites infection, partially due to the uncompetitive inhibition on CYP3A. The data presented in this paper indicate the promising tendency for CHR to be used as CYP 450 enzymatic inhibitor in coordination with ART-type anti-malarial drugs.

## Methods

### Chemicals and reagents

Artemisinin and internal standard artemether (ARM, Fig. 1b) were purchased from Chongqing Huali Konggu Co., Ltd. (purity >99.0 %, Chongqing, China). CHR (purity >98.0 %) was previously isolated and purified from the industrial wastes of ART (acetone layer) with about 1 % yield in the authors’ laboratory. Industrial waste was kindly gifted by Chongqing Huali Konggu Co. Ltd, and the voucher specimen of the waste has been deposited in the College of Pharmacy, Ningxia Medical University, for further references.

Six metabolic probes including phenacetin (PN), coumarin (CA), omeprazole (OMP), dextromethophan (DM), and chlorzoxazone (CLZ) were purchased from National Institutes for Food and Drug Control, China. Midazolam (MDZ) injection was provided by Jiangsu Enhua Pharmaceutical Co., Ltd (10 mg/2 mL). NADPH, EDTA, BCA kit, DTT, and Tris were of analytical grade and purchased locally. Acetonitrile and methanol (HPLC grade) were purchased from Fisher Chemicals (Fairlawn, NJ, USA).

Rat liver microsomes (RLM, Sprague–Dawley) were purchased from Wuhan Pulaite Medical and Technical Co. Ltd (M10011). Protein concentration was determined as 20 mg/mL by bicinchoninic acid (BCA) method using bovine serum albumin as standard.

### Instrumentation

**Fig. 2:**
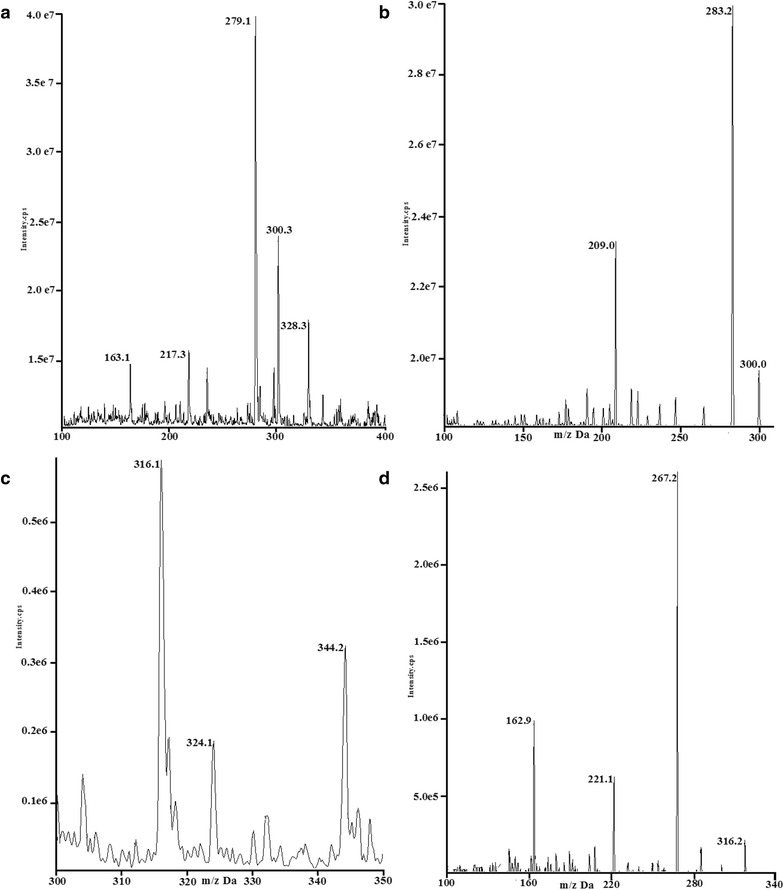
Collision-induced dissociation mass spectra for ART (**a** MS^1^ and **b** MS^2^) and ARM (**c** MS^1^ and **d** MS^2^). For experimental conditions see “Instrumentation”

The LC system for LC–MS/MS was Shimadzu Nexera UPLC LC-30A containing a binary LC-30AD pump, DGU-20A5 vacuum degasser, SIL-30AC autosampler and CTO-30A thermostat column compartment. An API 4000 triple quadrupole mass spectrometer (AB SCIEX, Foster, USA) with an electrospray ionization (ESI) operated in the positive ion mode was used for analysis. Quantification was performed using multiple reaction monitoring (MRM) for the transitions *m/z* 300. 1–209.0 for ARN and 316.2–163.0 for ARM (Fig. 2). The system was controlled by Analyst software version 1.5.1. Separation was performed on a Shimadzu XR-ODS C_18_ column (2.0 mm × 100 mm, 2.2 µm) with a Shimadzu ODS C_18_ security guard column (5 mm × 2.0 mm, 2.2 µm) maintained at 30 °C using a mobile phase containing acetonitrile and 0.1 % formic acid in 10 mM ammonium acetate (85:15, v/v) at a flow rate of 300 µL/min. The source temperature was maintained at 600 °C and the ESI source voltage was set at 5500 V. Collision gas pressure was 3 units and collision energy was 17 V.

The six enzymatic probe substrates were standardized by using Agilent 1200 (Agilent, USA) RP-HPLC system consisted of an on-line G1322A vacuum degasser, a G1311A quaternary pump, a G1329A injection valve (USA) with a sample loop of 20 μL, a G1314B UV–visible diode-array detector (DAD). A phenomenex C_18_ column (Synergi Hydro-RP 80A, 150 mm × 4.6 mm, 4 μm) was used as stationary phase with a flow rate of 1.2 mL/min at 30 °C. The isocratic mobile phase consisted of acetonitrile and purified water containing 1 % triethylamine and 0.02 M sodium dihydrogen phosphate (40:60 for PN, CA, DM, CLZ, and MDZ; 35:65 for OMP, v/v, PH = 3.5) was respectively used for assay of PN (wavelength: 250 nm), CLZ (282 nm), MDZ (230 nm), OMP (302 nm), CA (278 nm), and DM (202 nm).

### Stock solutions of chemicals

ART and CHR were separately suspended in 0.5 % carboxy methyl cellulose (CMC-Na) by sufficient emulsification to get the stock solution of 2 mg/mL strength and diluted to get the desired concentrations for each drug before it was administrated by the intramuscular injection or gavage perfusion. Master stock solutions for assay of blood concentration were individually prepared by dissolving ARN and ARM standards in acetonitrile at equivalent concentrations of 1000 μg/mL and were gradually diluted to 2 μg/mL by mobile phase for the preparation of calibration curve (0.2–200 ng/mL) and quality control (QC) samples (0.5, 10 and 160 ng/mL for ART), respectively.

For in vitro hepatic metabolic study, CHR and six enzymatic probes were separately prepared in methanol to strength of 1 mg/mL stock solutions and were diluted to desired concentrations by phosphate buffers (PBS, 0.1 M, PH = 7) before use. QC samples were solved in inactive RLM with three concentration levels (3.0, 28.0 and 89.0 for PN; 3.4, 34.2 and 100.0 for CA; 6.0, 29.5 and 118.0 for CLZ; 3.0, 15.0 and 49.0 for MDZ; 2.7, 13.5 and 43.2 for DM). A serial of RLMs in strength of 1.25, 1, 0.75, 0.5, 0.25 mg/mL were gained for optimization of protein concentration experiment by diluting the stock protein solution in strength of 20 mg/mL (stored at −80 °C). All other stock solutions were stored at 4 °C when not in use.

### Method validation

Calibration curves for artemisinin were constructed by weighted least-squares linear regression (1/*x*^2^) analysis of an eight-point calibration curve by plotting peak area of the analyte versus the peak area of the internal standard. The assay was validated through linearity, inter- and intra-day accuracy and precision, recovery, lower limits of quantification (LLOQ), stability, and matrix effect as usual.

Calibrations curves for six probe substrates in inactive RLM were obtained and the assay was evaluated through linearity, inter- and intra-day precision and accuracy, and recovery.

### Pharmacokinetics study

Male SD rats (200–240 g) were supplied by Laboratory Animal Center of Ningxia Medical University (Grade II, Certificate No. SCXY 2011-0001). Animals were acclimatized in environmentally controlled breeding cages for at least 3 days before being used and were provided with standard laboratory food and water and were fasted for 12 h prior to the study. Water was freely available for rats during experiments. The experimental protocol was approved by the University Ethics Committee. All procedures involving animals were in accordance with the Regulations of the Experimental Animal Administration, State Committee of Science and Technology, People’s Republic of China. 20 rats were randomly divided into 4 groups (*n* = 5). Group A was the control vehicle (ART alone) and group L, M, and H were the combination groups with CHR in three fixed ratios (1:1, 1:2, and 1:4). The suspension of CHR was continuously administered (each time per day) to rats in group L, M, and H by gavage at doses of 5, 10, 20 mg/kg for 3 days, respectively. Group A was orally given by 0.5 % CMC-Na solution once per day for 3 days. On day 3, an intramuscular injection of ART (5 mg/kg) was subsequently administered 1 h after CHR or CMC-Na solution pre-exposure. Blood samples (500 µL) were collected into centrifuge tube with heparin at 0, 10, 20, 30, 60, 90, 120, 240, 360, 480, 600, 720, and 1440 min from the orbit after the administration of ART, respectively. Two hours later, animals took food and water freely to replenish.

Plasma samples were separated by centrifugation 3500*g* for 10 min and 200 µL plasmas were stored at −20 °C until analysis. To 100 μL blank plasma, 50 μL ARM working solution (IS, 2 μg/mL), 100 μL methanol aqueous solution (50:50, v/v), and a standard solution or quality control (QC) solution were added. After vortex mixing for 1 min, the sample was extracted with 3 × 3 mL of methyl tert-butyl ether with gentle shaking for 10 min followed by centrifugation at 3500*g* for 10 min. The methyl tert-butyl ether extracts were combined and evaporated to dryness by a Pressure Gas Blowing Concentrator (multivap nitrogen evaporation system 118, Organomation Co., Ltd, USA) at 40 °C. The residue was reconstituted in 100 μL methanol and was filtered through a 0.22 µm nylon filter. Aliquots (10 μL) of the solution were injected onto the LC–MS analysis.

### In vivo anti-malarial efficacy

Female Kunming mice (18–22 g) for the experiment of in vivo chemo-suppressive study were purchased from Animal Center in Academy of Military Medical Science (Grade II, Certificate No. SCXK 2002-001). Animals were divided into eight groups (*n* = 10) including placebo group (normal saline only), ART alone (5 mg/kg), CHR alone (5, 10 and 20 mg/kg), and ART-CHR combination groups (1:1, 1:2, and 1:4). Two hours after being inoculated, they were administrated in the same delivery route and the same dosage of ART and CHR as pharmacokinetic study.

Stocks of the malaria parasite *P. berghei* K173 strain (chloroquine sensitive) were continuously maintained in the Microbiology Laboratory of Academy of Military Medical Sciences, China, and alternately stored by liquid nitrogen cryopreservation and blood subinoculation in mice. Approximate one million infected erythrocytes were inoculated into the enterocoelia of each mouse before use.

Twenty-four hours after the last dosing, the blood was collected from caudal vein of mice. Thin blood smear slides were air-dried, methanol-fixed, and stained in Giemsa for 40 min. The Giemsa-stained slides were examined for counting the number of parasites in random three microscopic fields, equivalent to over 200 erythrocytes each field at 1000× magnification. Percent parasitaemia (%) was calculated through dividing the number of total red blood cells by that of infected red blood cells. Percentage inhibition (%) for each drug was calculated relative to placebo. Reproducibility of counts was checked by two other readers to maintain the quality control.

### In vitro rat liver microsome experiment

The incubation system included 40 μL of RLM (protein concentration was optimized as 1 mg/mL for PN; 0.75 mg/mL for OPZ; 0.5 mg/mL for CA, DM, CLZ, and MDZ), 5 μL of substrate solution (substrate concentration was optimized as 25 μM for PN; 5 μM for CA; 20 μM for OPZ; 10 μM for DM; 15 μM for CLZ; 5 μM for MDZ), and NADPH-regenerating system (1.3 mM NADP^+^, 3.3 mM G-6-P, 3.3 mM MgCl_2_, 4 U/mL G-6-P-D) and a test compound (CHR, 0–50 μM). Total reaction volume was complemented to 200 μL by PBS. After pre-incubating for 3 min at 37 °C, the reaction was initiated by the addition of NADPH solution (incubation time was optimized as 45 min for PN; 60 min for CA, 15 min for OPZ; 20 min for DM; 90 min for CLZ; 15 min for MDZ). 200 μL ice acetonitrile was added as stop reagent. After a vortex for 3 min and centrifugation for 10 min at 14,000*g*, an aliquot of 20 μL supernatant was injected into the HPLC system at an interval of 10 min. The amount of methanol in diluted concentrations (<1 %) had no effect on liver microsomes. All the testing groups were in three replicates (*n* = 3). Under the optimized condition, V_max_, Michaelis constant (*K*_*m*_), enzyme activity (E%), and IC_50_ values were obtained.

### Inhibitory type determination

Incubation systems including 1.0 mg/mL RLM, PN (12.5, 25.0, and 50.0 μM), NPDPH, and CHR (0, 2, 4, 8 μM) for 1A2 or 0.5 mg/mL RLM, MDZ (2.5, 5.0, 10.0 μM), NPDPH, and CHR (0, 2, 4, 8 μM) for CYP3A were employed as above (*n* = 3). A serial of Lineweaver–Burk plots by 1/[S] versus 1/V were prepared individually under different concentrations of CHR. The location of intersection point for the linear lines decided the type of inhibition. Namely, if the point lied in Y axis it was competitive inhibition and if it lied in X axis it was non-competitive inhibition. Finally, if the linear lines were paralleled, it was uncompetitive inhibition. Concomitantly, inhibition constant (*K*_*i*_) was calculated when a second plotting was given by intercepts of the four lines versus [I] (inhibitor concentration).

### Data analysis

Pharmacokinetic parameters were computed from plasma concentration–time profiles of ART by software DAS 3.0. Statistical significance of differences was analyzed by SPSS 16.0 statistical software with a significance level of *P* < 0.05 and 0.01.

## Results and discussion

### UPLC-MS/MS method for ART

**Fig. 3:**
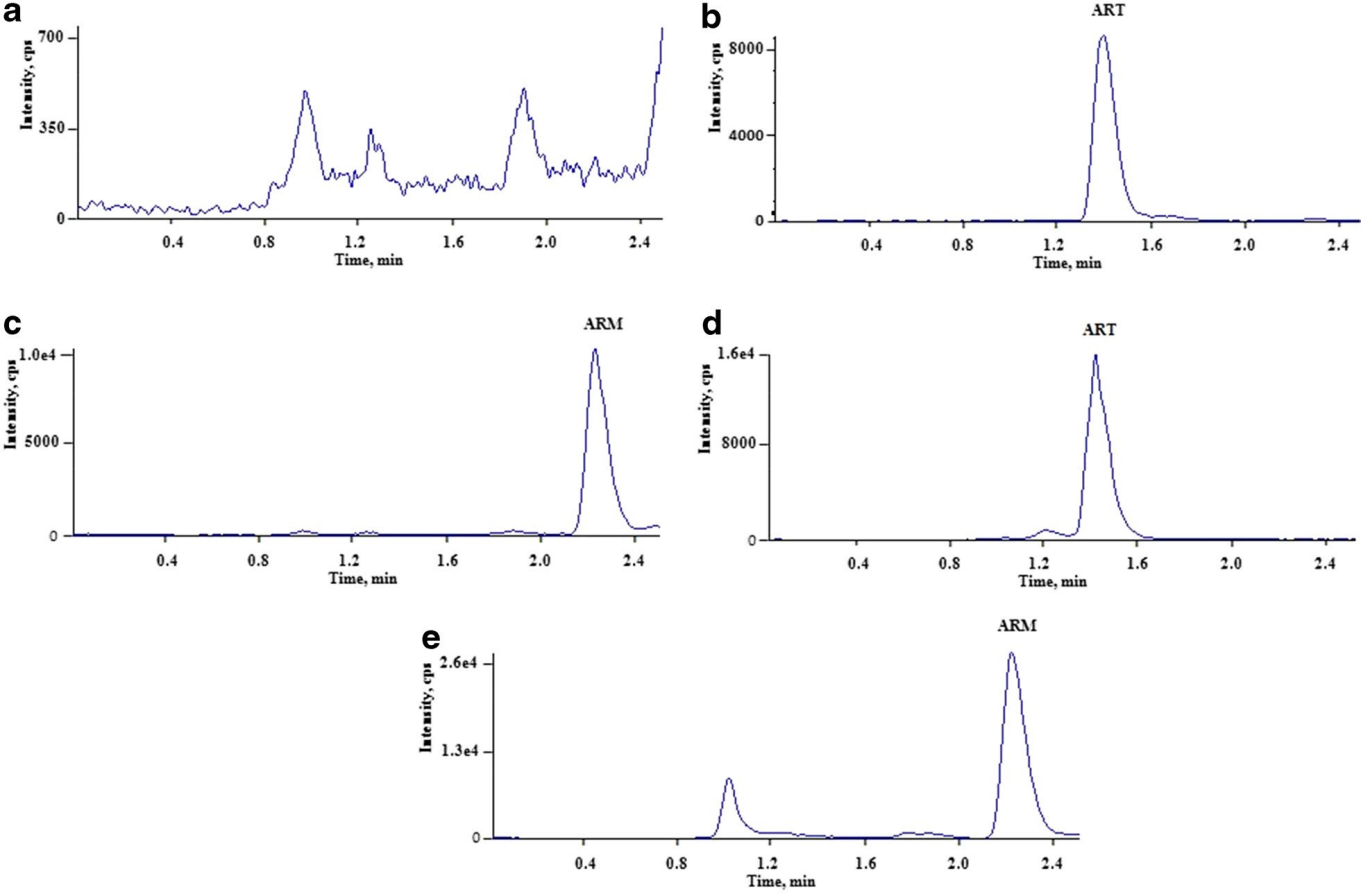
Representative full-scan chromatograms of (**a**) blank plasma, blank plasma spiked with ART (**b**) and ARM (**c**, IS), and a study sample containing ART (**d**) and ARM (**e**) after administration for 45 min

The electrospray ionization mass spectra for ART and ARM were shown in Fig. 2a (MS^1^ for ART), b (MS^2^ for ART), c (MS^1^ for ARM), and d (MS^2^ for ARM). Under optimized UPLC conditions, ART and ARM were eluted within 2.5 min (Fig. 3b, c). Blank plasma showed no significant interfering peaks at the retention times of each analyte (Fig. 3a). The calibration curve of ART was linear over the concentration range of 0.2–200.0 ng/mL (*y* = 0.0336*x* + 0.0259, *r* = 0.9965, w = 1/*x*^2^) with LLOQ 0.2 ng/mL (RSD% = 3.91, RE% = −5 %, *n* = 5). Inter- and intra-day precision and accuracy, stability, recovery, and matrix effect data were showed in Table 1. The precision and accuracy of this method indicated that all coefficients of variation at each concentration level are below 10 %. The normalized matrix effects of ART at low, middle, and high concentrations were close to 1 with a low variation in accordance with international guidelines [20]. ART was found stable in plasma for at least 6 and 24 h at ambient temperature before and after treatment and was also stable during three freeze/thaw cycles.

**Table 1:** Method validation for quantification of ART in rat plasma

QC (ng/mL)	Precision and accuracy (%)		Stability (RE%)				Recovery (%)	Matrix effect (*n* = 5) Mean (%)/RSD%
	Inter-day RSD/RE	Intra-day RSD/RE	Pre-T		Post-T	Freeze–thaw for three times		
			RT 6 h	RT 24 h	RT 24 h			
0.5	8.51/1.24	6.57/1.24	−4.00	−2.00	2.00	2.00	57.30	98.0/1.70
10	1.41/6.75	6.67/−1.90	3.20	−0.80	−8.20	6.80	73.30	88.0/2.70
160	2.97/7.81	6.99/0.67	−6.25	−3.00	−4.38	4.38	68.90	66.0/3.10

*RT* room temperature, *Pre-T* Pre-treatment, *Post-T* post-treatment

### HPLC–UV method for six probe drugs

Six probe drugs were well analysed within 10 min by HPLC–UV method without interference. Standard curves equations of six substrates in inactive RLM were displayed in Table 2. Details of method validation data were elaborately arranged in Table 3a, b in different strength of QC samples.

**Table 2:** Standard curves equation of six probe substrates in inactive RLM

Probe substrates	Calibration curves	*R* value	Linear range (μM)
PN	*Y* = 9.486*X* + 0.3485	0.9999	0.5–111
CA	*Y* = 6.730*X* + 0.7809	0.9999	0.7–137
OPZ	*Y* = 4.148*X* + 2.1741	0.9999	0.5–141
DM	*Y* = 24.868*X* + 5.1424	0.9999	0.5–54
CLZ	*Y* = 20.918*X* + 1.257	0.9999	1.0–295
MDZ	*Y* = 36.365*X* − 16.163	0.9999	0.8–63

**Table 3:** Method validation for quantification of PN, CA, OPZ, DM, CLZ, and MDZ in inactive RLM

QC (μM)	PN				QC (μM)	CA				QC (μM)	OPZ			
	Precision and accuracy (%)		Average recovery (%, *n* = 5)			Precision and accuracy (%)		Average recovery (%, *n* = 5)			Precision and accuracy (%)		Average recovery (%, *n* = 5)	
	Intra-	Inter-	Absolute	Method		Intra-	Inter-	Absolute	Method		Intra-	Inter-	Absolute	Method
a														
3.0	1.19	8.87	106.72 (1.77)	97.17 (1.19)	3.4	0.46	6.64	112.49 (1.12)	113.17 (0.46)	2.8	2.80	2.67	93.45 (7.79)	77.96 (6.02)
28.0	1.44	3.08	99.12 (3.05)	93.84 (3.30)	34.2	4.15	4.09	97.86 (2.92)	72.37 (4.15)	14.5	0.61	0.78	99.98 (3.70)	94.30 (1.52)
89.0	7.25	2.32	97.24 (1.22)	87.12 (1.42)	100.0	6.12	4.33	101.32 (6.68)	94.97 (6.12)	58.0	1.67	1.64	97.01 (0.32)	100.16 (0.65)

QC (μM)	CLZ				QC (μM)	MDZ				QC (μM)	DM			
	Precision and accuracy (%)		Average recovery (%, *n* = 5)			Precision and accuracy (%)		Average recovery (%, *n* = 5)			Precision and accuracy (%)		Average recovery (%, *n* = 5)	
	Intra-	Inter-	Absolute	Method		Intra-	Inter-	Absolute	Method		Intra-	Inter-	Absolute	Method
b														
6.0	1.26	3.83	94.74 (3.00)	94.48 (0.92)	3.0	3.87	5.29	98.51 (0.9)	100.50 (0.37)	2.7	11.92	10.50	81.50 (12.15)	104.70 (11.92)
29.5	1.31	4.90	97.14 (1.05)	95.31 (0.58)	15.0	1.93	3.36	100.07 (9.05)	107.18 (1.06)	13.5	5.56	8.56	96.75 (6.46)	98.33 (5.56)
118.0	2.96	1.97	102.60 (3.26)	97.12 (1.90)	49.0	0.17	2.53	93.42 (4.47)	98.79 (4.43)	43.2	4.43	2.37	99.96 (3.03)	94.45 (2.26)

The numbers in the brackets represented RSD%

### Pharmacokinetics parameters for ART in absence and presence of CHR

**Fig. 4:**
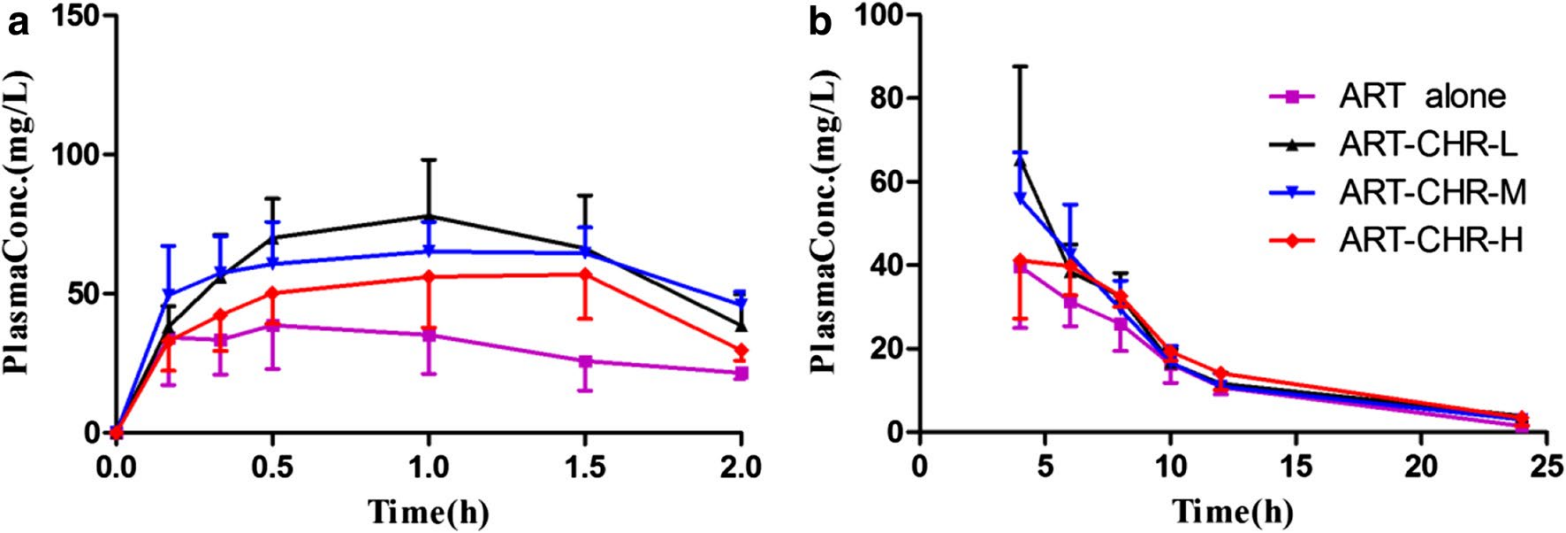
Mean (±SD) plasma concentration–time profiles of ART (*n* = 5) in ART alone (*square points*), ART-CHR-L (*triangular points*), ART-CHR-M (*inverted triangular points*), and ART-CHR-H (*diamond points*). **a** *before* 2 h, **b** *after* 2 h

Plasma concentration–time profiles of ART, in untreated rats and in pre-treated rats with different combinatory doses of CHR, were shown in Fig. 4a, b. Corresponding pharmacokinetic parameters were reported in Table 4. This study has demonstrated that the AUC_0-t_ of ART in ART-CHR-L (1:1), ART-CHR-M (1:2), and ART-CHR-H (1:4) groups, respectively, increased 1.44-, 1.40-, and 1.29-fold compared with ART alone (*P* < 0.01) without dose–response manner (*P* > 0.05 among the combination groups). The C_max_ in ART-CHR-L (1:1) and ART-CHR-M (1:2) group increased 1.64- and 1.65-fold versus control (*P* < 0.05) while no increase was found in ART-CHR-H (1:4) group. The *t*_1/2_ in ART-CHR-M (1:2) group enhanced 1.68 folds compared with ART alone (*P* < 0.05). It showed that oral co-administration of CHR in combinatory ratio of 1:2 prior to intramuscular injection of ART achieved a longer *t*_1/2_, a higher AUC and C_max_, and lower CLz than ART alone.

**Table 4:** Plasma concentration–time profiles of ART with or without CHR (*n* = 5)

Ratio	*t*_1/2_ (h)	AUC_0–t_ [mg/(L h)]	C_max_ (mg/L)	CLz (L/h/kg)
ART alone	3.62 ± 0.86	390.58 ± 32.46	51.58 ± 9.47	0.013 ± 0.001
ART-CHR-L (1:1)	3.89 ± 1.93	562.20 ± 101.50**	84.76 ± 19.52*	0.009 ± 0.002*
ART-CHR-M (1:2)	6.07 ± 2.04*^,#^	547.68 ± 76.59**	85.22 ± 29.56*	0.009 ± 0.001**
ART-CHR-H (1:4)	3.28 ± 0.77	505.21 ± 54.88**	63.16 ± 16.50	0.010 ± 0.001**

Data are mean values ± SD in five rats

Significantly different from ART alone: * *P* < 0.05, ** *P* < 0.01

Significantly difference from ART-CHR-H: ^#^ *P* < 0.05

### Synergic anti-malarial efficacy of ART in presence of CHR

The results showed in Table 5 suggested that parasitaemia (%) in ART alone, ART-CHR-L, ART-CHR-M, and ART-CHR-H groups significantly decreased 1.59, 2.23, 2.53, and 2.01 folds (*P* < 0.05) compared with placebo, whereas no significance was observed when CHR was solely used (*P* > 0.05). CHR, therefore, has no anti-malarial effect itself. Only in ART-CHR-M group, parasitaemia (%) and inhibition (%), respectively, achieved 1.59-fold reduction (*P* < 0.05) and 1.63-fold augment compared with ART alone. The result completely conformed to the pharmacokinetics study.

**Table 5:** In vivo antimalaria pharmacodynamic effect of ART in combination with CHR

Groups	Dosage (mg/kg)	Parasitemia (%, *n* = 10)	*P* value		Inhibition (%, *n* = 10)
			placebo	ART alone	
Placebo	0	22.15 ± 6.25	–	–	
CHR-L	5	19.74 ± 13.33	0.62	–	–
CHR-M	10	17.19 ± 5.45	0.05	–	–
CHR-H	20	18.32 ± 8.86	0.25	–	–
ART alone	5	13.95 ± 7.89*	0.01	–	37.06
ART-CHR-L	5 (1:1)	9.95 ± 5.36*	2.73 × 10^−5^	0.07	55.10
ART-CHR-M	10 (1:2)	8.75 ± 4.25*^,#^	2.16 × 10^−6^	0.04	60.51^#^
ART-CHR-H	20 (1:4)	11.00 ± 4.98*	2.72 × 10^−5^	0.18	50.36

* *P* values of drugs in relation to placebo: *P* < 0.05

^#^ *P* values of ART-CHR combination groups in relation to ART alone: *P* < 0.05

### Inhibition of CHR on CYP450 enzymes

*K*_*m*_ values for PN, CA, OPZ, DM, CLZ, and MDZ were calculated to be 30.88, 8.90, 24.07, 28.94, 19.97, and 7.88 μM with V_max_ 0.14, 0.12, 0.58, 0.20, 0.18, 0.81 nmol/min/mg proteins, respectively. The working substrate concentrations for PN, CA, OPZ, DM, CLZ, and MDZ, therefore, were confirmed to be 25, 5, 20, 10, 15, and 5 μmol L^−1^ (lower than *K*_*m*_).

**Fig. 5:**
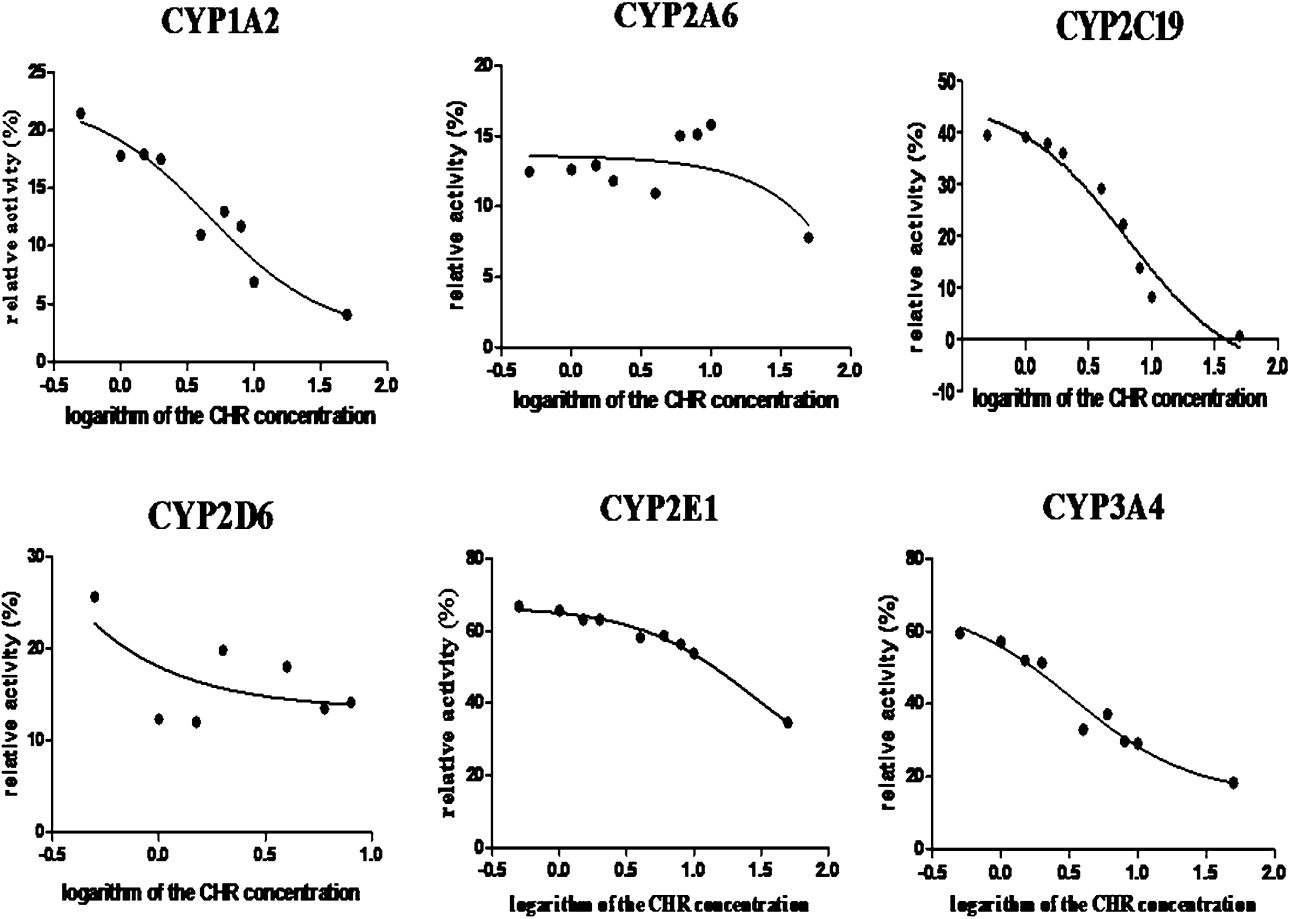
Inhibition curves of CHR on CYP450 isoforms

Graphs showed in Fig. 5 and data in Table 6 demonstrated that CHR has a significant inhibition (*P* < 0.01) on CYP1A2 and CYP2C19 in a concentration-dependence manner and enzyme activity (%) decreased from (21.64 ± 1.05) % to (4.05 ± 1.41) % for CYP1A2, and from (41.98 ± 0.35) % to (0.60 ± 0.11) % for CYP2C19 when CHR concentration increased from 0 to 50 μM. As for CYP2E1, CHR had a significant inhibition in a dose–response manner only after CHR concentration exceeded 4 μM (*P* < 0.05). Most interestingly, 0.5 μM of CHR significantly increased the enzyme activity (%) of CYP3A from 48.27 ± 0.93 (0.0 μM) to 59.44 ± 2.55 (*P* < 0.01), and it slowly decreased to 57.15 ± 0.93 (*P* < 0.01), 52.02 ± 3.07 (*P* > 0.05), and 51.37 ± 0.84 (*P* > 0.05) after CHR concentration added from 1.0 to 2.0 μM. An inhibition of CHR on CYP3A subsequently (*P* < 0.01) emerged after the concentration exceeded 4 μM as well as CYP2E1. No impact of CHR on CYP2A6 and CYP2D6 were found in this paper (*P* > 0.05).

**Table 6:** Effects of CHR on cytochrome P450 isoforms in rat liver microsomes in vitro

CHR/μM	Enzyme activity (%)					
	1A2	2A	2C19	2D6	2E1	3A
0	21.64 ± 1.05	14.45 ± 8.11	41.98 ± 0.35	19.25 ± 11.87	69.82 ± 7.31	48.27 ± 4.71
0.5	21.46 ± 2.83*	12.47 ± 1.91	39.56 ± 1.35**	25.62 ± 3.56	66.93 ± 1.82	59.44 ± 2.55**
1.0	17.80 ± 3.07**	12.62 ± 1.35	38.92 ± 0.56**	12.34 ± 12.08	65.76 ± 1.72	57.15 ± 0.93**
1.5	17.93 ± 1.15**	12.92 ± 0.86	37.64 ± 2.31**	12.03 ± 6.33	63.23 ± 2.40	52.02 ± 3.07
2	17.50 ± 0.58**	11.82 ± 1.76	36.14 ± 2.19**	19.81 ± 0.64	63.22 ± 2.06	51.37 ± 0.84
4	10.96 ± 3.56**	10.94 ± 6.85	29.22 ± 1.06**	18.03 ± 4.02	58.21 ± 5.63*	32.94 ± 2.87**
6	12.99 ± 1.56**	15.03 ± 0.33	21.40 ± 3.06**	13.49 ± 10.99	58.79 ± 2.29*	37.18 ± 0.46**
8	11.72 ± 2.81**	15.12 ± 0.96	13.84 ± 0.49**	14.16 ± 7.44	56.44 ± 5.63*	29.70 ± 4.12**
10	6.89 ± 1.56**	15.82 ± 1.62	8.30 ± 1.04**	ND	53.81 ± 2.02*	29.08 ± 3.76**
50	4.05 ± 1.41**	7.79 ± 0.95	0.60 ± 0.11**	ND	34.62 ± 6.25*	18.27 ± 14.55**

*ND* no detection

Compared with control group (no CHR)

* *P* < 0.05,** *P* < 0.01

IC_50_ values were computed to 4.61 μM for 1A2, 6.23 μM for 2C19, 3.38 μM for 3A, and 28.17 μM for 2E1 by GraphPad Prism 5.0 software. CHR is a moderate inhibitor on 1A2, 3A, and 2C19 (1 < IC_50_ < 10 μM) and a weak inhibitor on 2E1 (IC_50_ >10 μM) according to the research [21].

### Inhibitory type determination for 1A2 and 3A

**Fig. 6:**
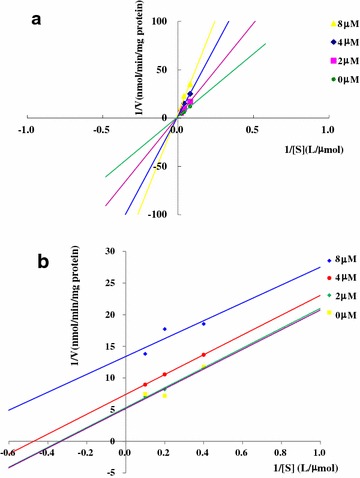
Inhibition types determination for 1A2 (**a**) and 3A (**b**)

Figure 6a, b showed that the type of CHR as an inhibitor on CYP1A2 belonged to non-competitive (intersection point lied in X axis) and that on CYP3A was uncompetitive inhibition (parallel lines). *K*_*i*_ was determined to 4.07 μM for CYP1A2 (*y* = 33.221*x* + 135.2, *r* = 0.9904) and 3.71 μM for CYP3A (*y* = 0.5416*x* + 2.012, *r* = 0.9600).

**Fig. 7:**
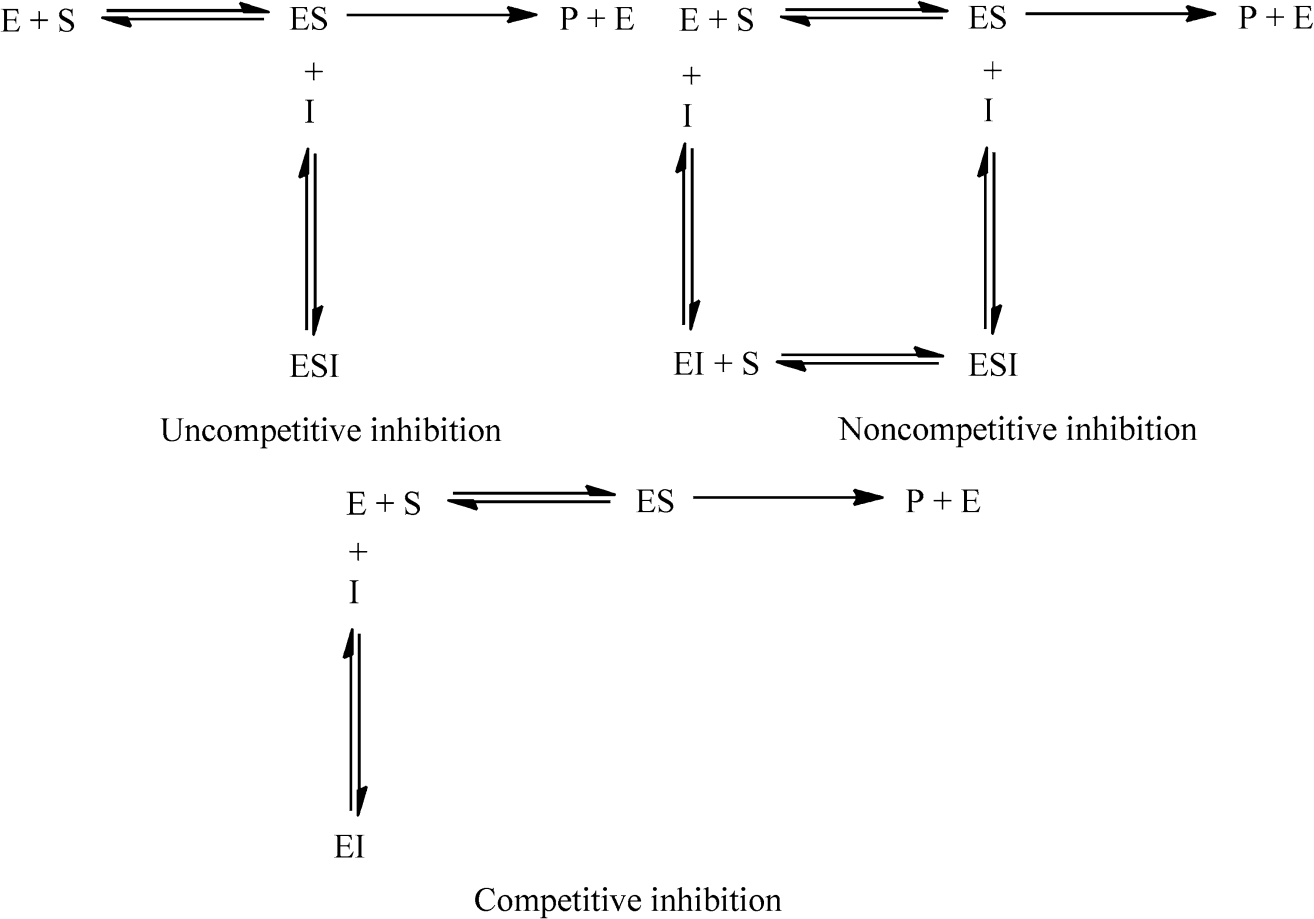
Diagrammatic sketches showed that the difference among uncompetitive, noncompetitive inhibition, and competitive inhibition

Inhibition of inhibitors on enzymes has two types, namely irreversible and reversible inhibition. The latter includes competitive, noncompetitive, uncompetitive and mixed competitive inhibition (showed in Fig. 7). The dynamic characteristic of uncompetitive inhibition (substrate is indispensable for the inhibition) is that both V_max_ and *K*_*m*_ decreased when inhibitor concentration increased. This type of inhibition is infrequency in single substrate catalysis of enzyme. This study demonstrated that CHR acted as an on uncompetitive inhibitor of CYP3A activity and as a non-competitive inhibitor of CYP1A activity. The results are in accordance with the literatures [22, 23]. Especially, the fact that CHR inhibited CYP3A in rat liver microsome in an uncompetitive manner might be meaningful for us to partly explain why CHR has a partial dose-dependent manner in pharmacokinetics and anti-malarial efficacy studies. More interestingly, initially increased enzyme activity of CYP3A under a low concentration of CHR treatment was observed in the inhibition experiment using RLM. The authors speculate that the phenomenon might be closely related to the uncompetitive inhibition type of CHR on CYP3A. CHR exclusively bonded to enzyme-substrate complex (ES) instead of enzyme itself; therefore, when inhibitor is added into the reaction, the equilibrium reaction will shift to the product generation direction, which enhances the production of ES complex instead. When CHR concentration is very low, ES complex may not be fully bonded. The surplus will continue to be decomposed to enzyme and product by enzymolysis. So the enzyme activity initially increased a little compared with the control. As CHR concentrations increased, however, the activity slowly decreased and then inhibition effect was finally observed due to the binding between CHR and ES complex and therefore the hindering of the enzymolysis. The increased activity might, therefore, not be activated by induction of CHR on CYP3A but possibly depends on the shifting of equilibrium reaction. Further research should be carried out to demonstrate the hypothesis.

## Abbreviations

UPLC: ultra-high performance liquid chromatography
MS: mass spectrometry
MS/MS: tandem mass spectrometry
CHR: chrysosplenetin
ART: artemisinin
ARM: artemether
ACT: artemisinin-based combination therapy
NADPH: nicotinamide adenine dinucleotide phosphate
BCA: bicinchoninic acid
DTT: dithiothreitol
Tris: tris(hydroxymethyl)metyl aminomethane
